# Supplementary material for: Live cell imaging of single genomic loci with quantum dot-labeled TALEs
Source: Nat Commun. 2017 May 8;8:15318. doi: 10.1038/ncomms15318 (PMC5424152; doi:10.1038/ncomms15318)
Supplement: Supplementary Information — Supplementary Figures [file ncomms15318-s1.pdf]

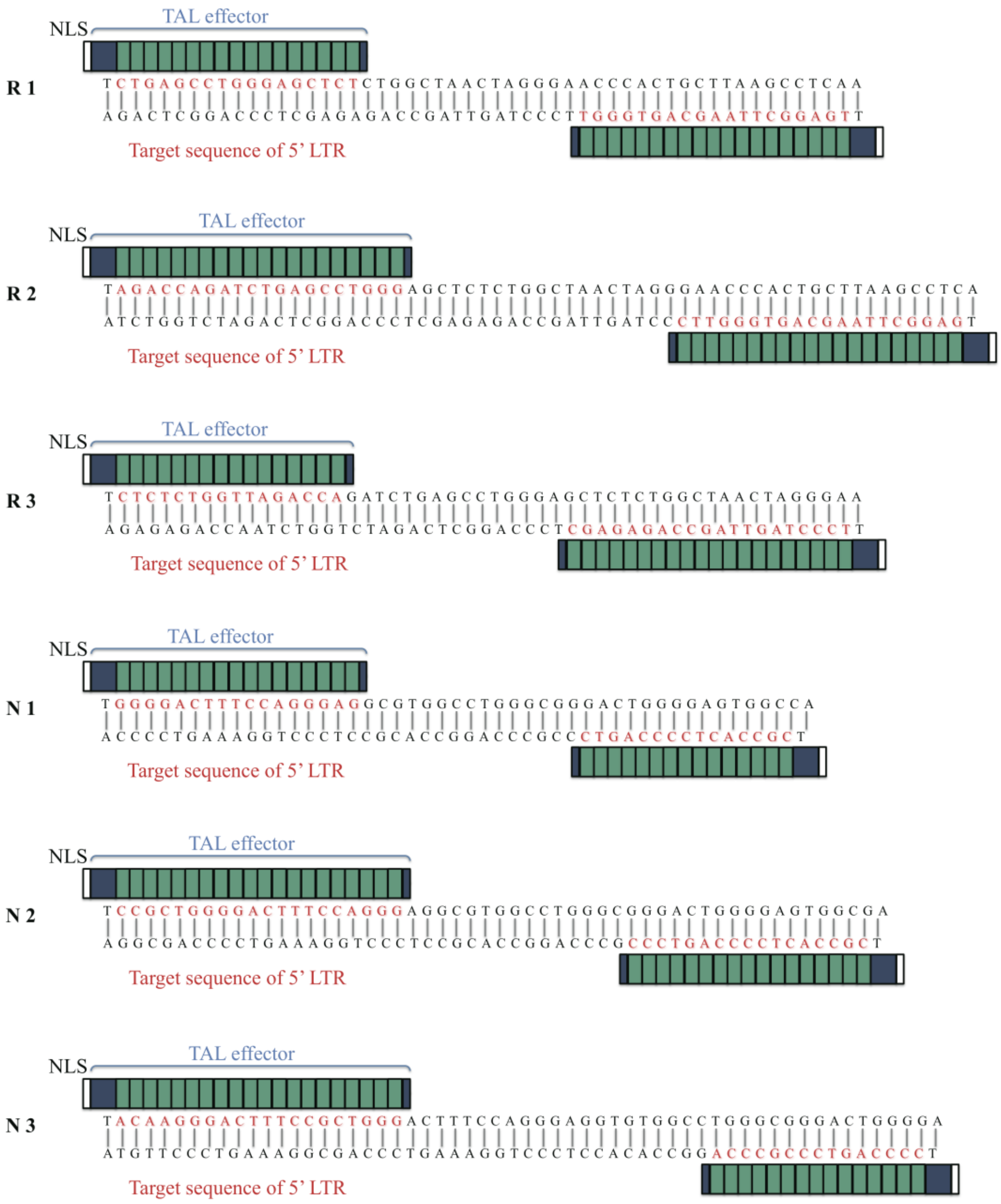

Supplementary Figure 1. Six pairs of TALE for target sequences of LTR. To validate the binding activity of TALEs, we designed six pairs of TALEs against different sequences. Schema depicted pairs of TALEs and their target sequences.

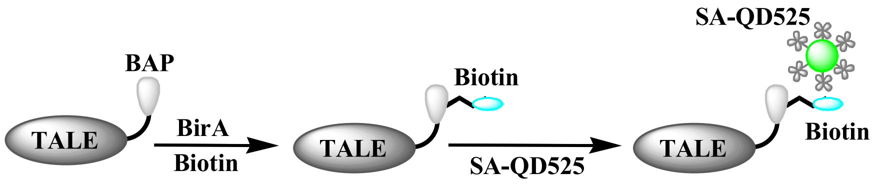

Supplementary Figure 2. Schematic diagram of fluorescence labeling of TALE N2-R with green QDs. Firstly, a 15-amino acid peptide (BAP tag) was fused to TALE N2-R and specifically biotinylated by biotin ligase (BirA) in live cells. Further addition of streptavidin-conjugated QD525 (SA-QD525) allows the labeling of TALE N2-R with QD525 because of the interaction of biotin and streptavidin.

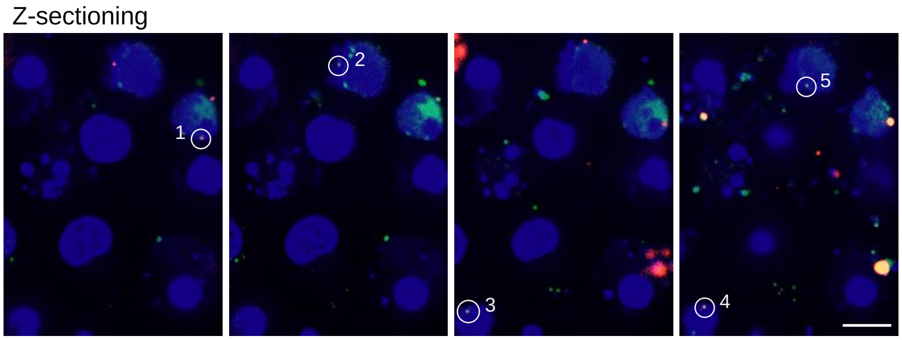

Supplementary Figure 3. Imaging of single HIV provirus loci in live U1 cells. Optical sections of colocalization signals were taken along the z-axis in multiple cells. The colocalization signals are highlighted by white circles (Scale bar, 15 µm).

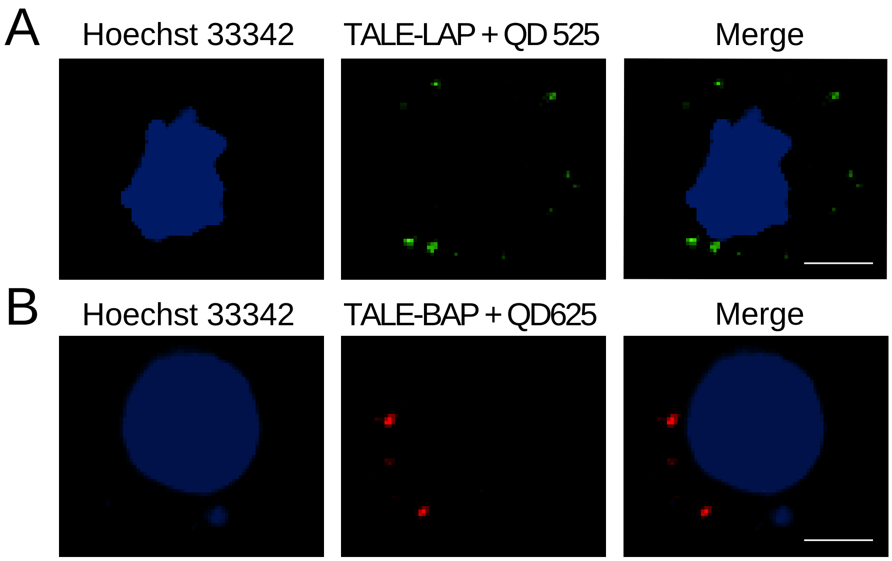

Supplementary Figure 4. (A) Images of TALE-LAP and SA-QD525 being transfected into a live cell. (B) Images of TALE-BAP and Tz1-QD625 being transfected into a live cell. Scale bar, 5 μm.

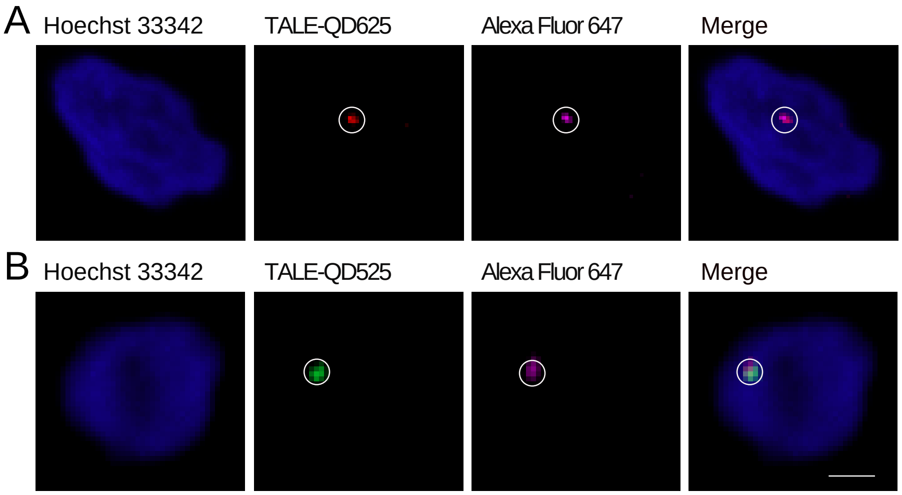

Supplementary Figure 5. (A) Colocalization imaging of TALE-LAP-QD625 with HIV specific FISH using Alexa Fluor® 647-tagged DNA probes. (B) Colocalization imaging of TALE-BAP-QD525 with HIV specific FISH using Alexa Fluor® 647-tagged DNA probes. Scale bar, 5 μm.

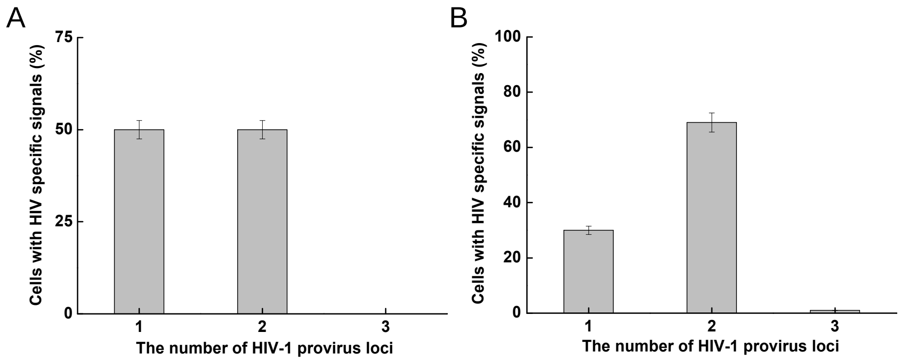

Supplementary Figure 6. (A) Analysis of the number of HIV provirus loci in U1 cells (n=200) with HIV specific colocalization signals of TALE-QDs. (B) Analysis of the number of HIV provirus loci in U1 cells (n=200) with HIV specific DNA-FISH fluorescent signals. Error bars denote s.d.; n=3.
